# The Coronal Alignment of Lower Limbs in the Adolescent Football and Ice Hockey Players

**DOI:** 10.1007/s43465-023-01061-8

**Published:** 2023-12-09

**Authors:** Nik Žlak, Zmago Krajnc, Aljaž Merčun, Matej Drobnič, Alan Kacin

**Affiliations:** 1 Department of Orthopaedic Surgery, University Medical Centre Ljubljana, Zaloška ulica 9, 1000 Ljubljana, Slovenia; 2 Chair of Orthopaedics, Faculty of Medicine, University of Ljubljana, Ljubljana, Slovenia; 3 Department of Orthopaedic Surgery, University Medical Centre Maribor, Maribor, Slovenia; 4 Chair of Orthopaedics, Faculty of Medicine, University of Maribor, Maribor, Slovenia; 5 Department of Physiotherapy, Faculty of Health Sciences, University of Ljubljana, Ljubljana, Slovenia

**Keywords:** Coronal alignment, Lower limbs, Varus, Bowlegs, Football, Ice hockey, Adolescent, Male

## Abstract

**Background:**

To investigate the influence of sport-specific activities on coronal axial alignment of the lower limbs in adolescent football and ice hockey players.

**Methods:**

This cross-sectional study targeted healthy adolescent male football and ice hockey players with at least 3 years of sports participation. 90 football and 38 ice hockey players aged 12–16 years were divided into five age-matched subgroups. Coronal alignment of the lower limbs was determined by measuring the players' intercondylar or intermalleolar (ICD-IMD) distance with a custom-made calliper. In addition, their sports history was recorded. An age-matched comparison between the two sports groups was performed using the two-way model ANOVA and a multiple regression model for ICD-IMD was constructed. Results were additionally compared with age-matched data from the general population published in recent literature.

**Results:**

A statistically significant increase in ICD-IMD values (*p* < 0.05) was found between 12 (football 0 mm; ice hockey − 64 mm) and 16 years (football 340 mm; ice hockey 310 mm) in both sports groups. Results of regression analysis of pooled group data showed that ICD-IMD has low positive correlation (r = 0.407; r^2^ = 0.168; *p* < 0.05) with time of participation in sport, but no association with age of athletes at the start of their sport participation (r = − 0.018; r^2^ = 0.000; *p* > 0.05). There were no statistically significant differences between the two groups at any time point. Both sports groups showed a significant increase in ICD-IMD values (mean 198 mm) after the age of 14 compared to the general population.

**Conclusions:**

Participation in football and ice hockey is associated with a similar increase in ICD-IMD in the adolescent years in male athletes. The observed increase was higher in both groups of athletes than in their peers who do not regularly participate in sports.

**Level of Evidence:**

Level 4 (case series).

## Introduction

More and more children and young adults participate in a variety of sports every day [1, 2]. It is generally accepted that participation in sport improves overall health. However, it also increases the risk of musculoskeletal injuries [3, 4]. It is estimated that almost 10% of children are injured during sporting activities each year [5] and about 8% are forced to give up their sporting activities due to sports injuries [6].

In healthy children, the axes of the lower limbs change dynamically during the first and, to a lesser extent, the second decade of life [7, 8]. At birth, the coronal alignment of the knee is typically varus, but by the second year of life, varus begins to gradually rotate towards valgus, peaking between the ages of 3 and 5 years, followed by spontaneous correction by the age of 7 years to a physiological alignment of 1° valgus to 3° varus with ethnic and gender differences [7–11]. The development of bow legs (varus knee alignment above the physiological value of 3°) in late childhood and early teenage years is considered atypical, but in the majority of cases not pathological [3, 9, 12]. There are numerous factors that contribute to the development of lower limb varus, such as environmental conditions, sports activities, gender, obesity, metabolic disorders, vitamin D deficiency, etc. [9, 12].

Bones react strongly to mechanical stress during adolescence. According to the Heuter–Volkmann law, supra-physiological compression forces can stop the growth of the physe, while distraction can lead to its hyper-growth [13]. Since the medial compartment carries about 70% of the load on the knee [14], frequent rapid accelerations and decelerations, crossover cutting tasks, pivot shift movements and other movement patterns with strong abduction forces and weight-bearing loads can stop the growth of the proximal medial tibial physis and promote the development of a varus knee. The main shift towards varus alignment probably occurs during the growth spurt [15].

Varus malalignment alters lower limb biomechanics by shifting the centre of the mechanical axis medially, which increases the load on the medial knee compartment, increasing the risk of early-onset degenerative joint disease [12, 16, 17]. In addition, bowed legs predispose athletes to various overuse injuries, patellofemoral pain syndrome, meniscal lesions and medial tibial syndrome [3, 17, 18]. It has been clearly demonstrated that participation in sports is associated with an increased incidence of varus knee alignment in young athletes compared to peers who do not participate in sports [3, 8, 17, 19–21]. This is particularly true for young football players, which is probably due to the specific loading patterns in football [3, 8, 17, 20, 21]. The influence of other sports, e.g. ice hockey, on axial alignment of the lower limbs is less well studied. Nevertheless, some studies reported a trend towards increased varus knee alignment in children participating in weight-bearing sports [15, 22].

Football and ice hockey are two biomechanically completely different sports. Football involves running and kicking, with varus and internal rotation predominating in the hip, while ice hockey involves skating, with valgus and external rotation predominating in the hip. As both sports are common in our sub-alpine region and are played by a similar paediatric population, we decided to investigate and compare their effects on lower limb axial alignment.

The aim of our study was therefore twofold: (1) to analyse the development of lower limb axial deviations at the knee level in adolescent football and ice hockey players, and (2) to compare the results with data from the non-athletic population.

## Materials and Methods

### Study Design

The study was designed as a cross-sectional study and its protocol was approved by the National Medical Ethics Committee (Approvals Nos. 86/02/13 and 44/12/13). The study was conducted in accordance with the Declaration of Helsinki (1964). Signed informed consent was obtained from all participants and their parents.

### Subjects Data

The adolescent subjects were recruited from the male youth teams of two national premier league teams: the football club NK Maribor and the ice hockey team HK Olimpija Ljubljana. The medical and sporting history of the potential candidates was first recorded and checked. Participants were required to participate in regular sport for at least 3 years prior to recruitment, i.e. 3–5 training sessions per week plus competitions according to the age group programme. Individuals with a history of severe lower limb injury or surgery of any kind, known systemic musculoskeletal disorders, malformations and deformities or joint hyperlaxia were excluded. Based on the selection criteria, 90 football and 38 ice hockey players aged 12–16 years were included in the study. They were divided into 5 age groups: from 12 (≥ 12 and < 13) to 16 (≥ 16 and < 17) years.

### Measurements

Coronal alignment of the lower limbs was determined by measuring inter-condylar (ICD) and inter-malleolar (IMD) distances with a calliper [8, 10]. Subjects stood in a relaxed, upright position with both feet pointing straight forward, hips and knees maximally extended and touching the medial femoral condyles or medial malleoli. Positive ICD-IMD values indicate increased intercondylar distance (varus alignment), while negative ICD-IMD values indicate increased intermalleolar distance (valgus alignment). All measurements were performed alternately by two trained examiners and repeated three times for each participant. The technical details of the measurement are described in Krajnc et al. [23].

### Sample Size Estimation

ICD-IMD was defined as the primary outcome measure of the study. The minimum sample size estimate at a statistical power of ≥ 0.90 (β-error ≤ 10%) was calculated for the ICD-IMD interaction effect of the factorial ANOVA (age × sport) based on the standardised effect size and SD, both pooled from a pilot sample. Assuming a 20% interaction effect, the estimated minimum number of participants was 12 (Statistica, StatSoft Inc., Tulsa, Oklahoma, USA).

### Data Management and Statistical Analysis

Data are presented as means ± standard deviation (SD). The ICD-IMD values were first compared by two-way analysis ANOVA (age × sport) with repeated measures for one factor between age groups and sports. The statistics reported for the interaction of the factors are the *p*-value (α), the effective size (ηp^2^) and the statistical power (1 − β). In case of a significant interaction or effect of any of the factors, the pairwise post-hoc comparisons were performed using Tukey's test for honestly significant differences. The obtained ICD-IMD values were also compared with the values of the age-matched general population obtained by Witvrouw et al. [17] using the *t*-test for unpaired samples. The associations between the values of ICD-IMD and the duration of participation in sport and the age at which participation started were analysed with bivariate linear regressions, Pearson correlation coefficients (r) and coefficients of determination (r^2^). Associations were calculated for the pooled group data and separately for the football and ice hockey groups. The statistical significance level for all tests was set at *p* < 0.05. Statistical analysis was performed using Statistica software (version 12, StatSoft Inc., Tulsa, Oklahoma, USA).

## Results

The distribution of athletes among age groups was similar: in the 12-year-old group there were 19 footballers and 8 ice hockey players, in the 13-year-old group there were 20 footballers and 8 ice hockey players, in the 14-year-old group there were 19 footballers and 7 ice hockey players, in the 15-year-old group there were 15 footballers and 8 ice hockey players, and in the 16-year-old group there were 17 footballers and 7 ice hockey players. There was no significant difference between the sport groups in terms of age at initiation of sport participation (football = 7.1 ± 1.2 years; ice hockey = 6.8 ± 2.1 years; *p* = 0.553) and in terms of duration of participation in each sport (football = 6.8 ± 1.7 years; ice hockey = 5.7 ± 2.3 years; *p* = 0.668).

**Fig. 1:**
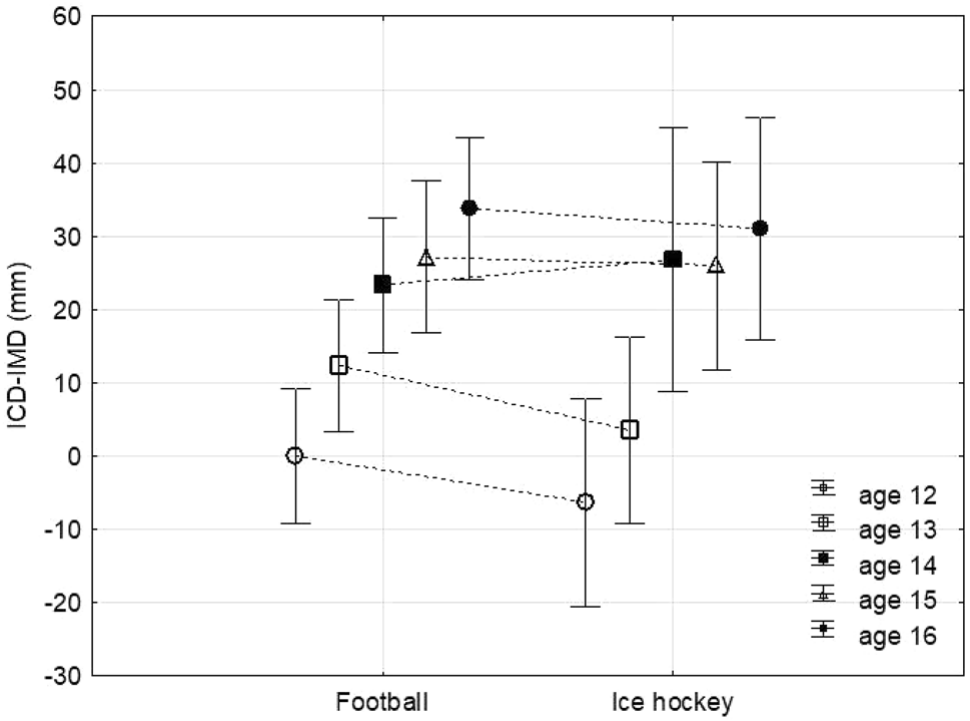
ICD-IMD values by sport and age subgroups (mean ± SD)

The interaction of the factors age and sport was not significant (*p* = 0.892; ηp^2^ = 0.009; 1 − β = 0.110), so there were no statistically significant differences between the two sport groups at any age. There was a highly significant effect of the age factor (*p* < 0.001; ηp^2^ = 0.281; 1 − β = 0.999) and a non-significant effect of the sport factor (*p* = 0.437; ηp^2^ = 0.005; 1 − β = 0.121). The results of the post hoc analysis showed a statistically significant increase (*p* < 0.05) in ICD-IMD values between 12 and 16 years (12 years; football 0 mm ± 162 mm; ice hockey − 64 mm ± 272 mm, 13 years; football 123 mm ± 203 mm; ice hockey 35 mm ± 167 mm, 14 years; football 233 mm ± 225 mm; ice hockey 268 mm ± 306 mm, 15 years; football 271 mm ± 233 mm; ice hockey 260 mm ± 109 mm, 16 years; football 340 mm ± 199 mm; ice hockey 310 mm ± 131 mm) in both sport groups (Fig. 1). Both sports groups showed a significant increase in ICD-IMD values compared to the general population; football players at age 13 (general population: − 75 mm ± 238 mm, football players: 123 mm ± 203 mm, *p* = 0.001) and ice hockey players at age 14 (general population 52 mm ± 291 mm; ice hockey players 268 mm ± 306 mm, *p* = 0.003).

Results of regression analysis of pooled group data showed that ICD-IMD has low positive correlation (r = 0.407; r^2^ = 0.168; *p* < 0.05) with time of participation in sport, but no association with age of athletes at the start of their sport participation (r = − 0.018; r^2^ = 0.000; *p* > 0.05). Regression analysis of football group showed that ICD-IMD has low positive correlation (r = 0.392; r^2^ = 0.154; *p* < 0.05) with duration of participation in sport, but no association with age at the start of sport participation (r = − 0.024; r^2^ = 0.000; *p* > 0.05). Likewise, regression analysis of ice hockey group showed that ICD-IMD has low positive correlation (r = 0.436; r^2^ = 0.190; *p* < 0.05) with duration of participation in sport, but no association with age at the start of sport participation (r = − 0.061; r^2^ = 0.004; *p* > 0.05).

## Discussion

The main findings of the study presented were that participation in football and ice hockey led to an increase in ICD-IMD values in adolescent boys aged 12–16 years. No statistically significant differences were found between the two sport groups at ay age. Both sports increased the development of varus alignment of the knee compared to peers who did not participate in sports.

Our results in adolescent football players are consistent with previous studies showing a shift in lower limb alignment towards varus of the knee at the time of the second growth spurt [8, 19–21]. In contrast, there is no published data on lower limb alignment in ice hockey players. We only found the study by Thijs et al. [3] reporting on lower limb alignment in field hockey players, whose movement patterns are somewhat similar to those of ice hockey and can therefore be used for comparison. They observed a similar knee varus pattern in various sports such as football, athletics, basketball, volleyball, tennis, badminton, squash and field hockey [3]. They reported a significantly greater intercondylar distance in athletic boys aged 13–15 years compared to peers not involved in sports (athletic ICD-IMD = 49 mm vs. nonathletic ICD-IMD = − 63 mm). Based on these results, they hypothesised that participation in weight-bearing sports contributes significantly to the development of bow legs. In our study, the average ICD-IMD in 13- to 15-year-old ice hockey players was 73 mm, confirming their hypothesis. A significant relationship between varus knee alignment and weight-bearing sports was also observed in a large study by De Cock et al. [15]. They demonstrated that all weight-bearing activities (tennis, volleyball, dancing, basketball, jogging) cumulatively contribute to the development of varus knee alignment, but did not investigate whether the duration of exercise was also a factor. As described by Basier et al. [24] and Schipplein et al. [25], repetitive movement patterns increase compression forces on the medial knee compartment, so it is reasonable to assume that the duration of participation in sport could play an important role. The results of our bivariate correlations showed that the duration of sport participation can explain about 16% of the increase in knee varus in football players and 19% in ice hockey players, while age at the beginning of sport participation showed no association. A more recent study by Insin and Melekoǧlu [21] also found a positive association between knee varus deformity and duration of football participation, but with a much higher correlation explaining up to 64% of the change in ICD-IMD value. The difference in the strength of the association could be due to the wider age range (10–18 years) and the larger study sample (n = 237). Interestingly, our data suggest that the association is stronger in ice hockey players, which does not support the hypothesis that kicking the ball is one of the mechanisms for increased varus formation in the knee in adolescent football players [8, 17].

As highly repetitive weight-bearing movement patterns are typical for both football and ice hockey, they could explain the increased varus formation in the knee observed in both sports in our study. In contrast, some other authors [8, 17] suggested that the increased forces acting on the medial knee compartment during repetitive ball kicking are the main mechanism for the increased varus formation in football players, neglecting the influence of other activities (increased adduction torque, rapid changes of direction, cutting tasks, etc.). If ball kicking were the main external factor for varus formation in the knee, one would expect more deformation of the dominant leg in football players. That this is not the case has already been clearly shown by Krajnc et al. [19] and Colyn et al. [22]. Krajnc et al. [19] demonstrated asymmetric involvement of the tibial growth plate after intensive training, resulting in changes in the medial tibial growth zone associated with attenuated growth on both legs, while Colyn et al. [22] found no difference in knee alignment between the stance and playing legs in football players.

Varus knee alignment as a result of ever increasing training intensity, decreasing age of children participating in sports at a competitive level, and premature focus on a single sporting activity predisposes athletes to various overuse injuries and syndromes [1, 3, 17, 18, 26]. It is well known that athletes, especially football players, have a higher prevalence of varus alignment of the knee, which has many negative consequences for knee health in the medium term [17, 18, 24, 27]. The injury incidence varies and depends on age, gender and sport. According to Caine et al. [28], hockey, American football and football (soccer) have the highest frequency of injury in boys, while football, basketball and gymnastics have the highest frequency of injury in girls. It has been described that athletes who participate in weight-bearing sports with high impact and torsional loads on the joints have an increased risk of knee injuries and early development of osteoarthritis [29, 30]. As football and ice hockey players have been observed to have a very similar tendency to develop varus alignment of the knee with age, they appear to have a similar increased risk of meniscal injury, patellofemoral pain, tibial stress syndrome and early development of osteoarthritis [18, 31, 32].

The following limitations of our methodology need to be considered. First, the sample size was small and we did not include a control group of non-athletic peers. However, we used previously published data from non-athletes in a comparable age range [3, 17], which clearly showed that both sport groups had higher ICD-IMD values at a given age. Secondly, standing height and leg length were not taken into account when comparing ICD-IMD values between age groups. Since we studied young players with active longitudinal bone growth, the observed increase in the absolute value of ICD-IMD may not be a pure representation of changes in varus/valgus position of the knee. The increase in ICD-IMD value could be a function of the increase in bone length and not the change in hip-knee-ankle angle. However, Cahuzac et al. [9] found no correlation between height and ICD-IMD distance, so absolute ICD-IMD values were used in most alignment studies [3, 7, 8, 15, 17, 21]. Thirdly, the measurement of intercondylar and intermalleolar distance cannot distinguish between possible asymmetries in the alignment of the left and right knee. Furthermore, there is a possibility of subjective bias when measuring ICD-IMD with a calliper. Therefore, in the presented study, great attention was paid to the standardisation of the measurement protocol and the measurements were repeated alternately by the two trained investigators. Krajnc and Drobnič [19, 23] had shown that the clinical measurement ICD-IMD values correlates well with the results of radiographs, therefore the method can be considered valid for population studies.

## Conclusions

Participation in both football and ice hockey was associated with an increase in ICD-IMD values in male adolescents. However, no statistically significant differences were found between the two groups at any age. The increase in ICD-IMD values was increased in both sport groups compared to their non-athletic peers. Our data reinforce the paradigm that the intensity and frequency of engagement in the primary sport discipline should be reduced in adolescent athletes during the period of accelerated growth and replaced by more diverse and less intensive physical activities.

## Data Availability

The data supporting the results of this study are available on request from the principal investigator, Dr. Alan Kacin (alan.kacin@zf.uni-lj.si).
